# Dental Students’ Educational Achievement in Relation to Their Learning Styles: A Cross-sectional Study in Iran

**DOI:** 10.5539/gjhs.v7n5p152

**Published:** 2015-02-24

**Authors:** Seyed Masoud Hosseini, Hamideh Amery, Ali Emadzadeh, Saber Babazadeh

**Affiliations:** 1 School of Nursing and Midwifery, Mashhad University of Medical Sciences, Mashhad, Iran; 2 School of Dentistry, Mashhad University of Medical Sciences, Mashhad, Iran; 3 School of Health, Hamedan University of Medical Sciences, Hamedan, Iran

**Keywords:** dental education, educational achievement, Kolb Learning Style

## Abstract

**Background and Objectives:**

In recent decades, many studies have been carried out on the importance of Kolb experiential learning theory (ELT) in teaching-learning processes and its effect on learning outcomes. However, some experts have criticized the Kolb theory and argue that there are some ambiguities on the validity of the theory as an important predictor of achievement. This study has been carried out on dental students’ educational achievement in relation to their dominant learning styles based on Kolb theory in Mashhad University of Medical Sciences (Iran).

**Methods:**

In a cross sectional study, Kolb Learning Style Inventory (LSI Ver. 3.1) as well as a questionnaire containing students’ demographic data, academic achievement marks including grade point average (GPA), theoretical and practical courses marks, and the comprehensive basic sciences exam (CBSE) scores were administered on a purposive sample of 162 dental students who had passed their comprehensive basic sciences exam. Educational achievement data were analyzed in relation to students’ dominant learning styles, using descriptive and analytical statistics including χ^2^, Kruskal-Wallis and two-way ANOVA tests.

**Results:**

The dominant learning styles of students were Assimilating (53.1%), Converging (24.1%), Diverging (14.2%) and Accommodating (8.6%). Although, the students with Assimilating and Converging learning styles had a better performance on their educational achievement, there was no significant relationship between educational achievement and dominant learning style (P≥0.05).

**Conclusion:**

Findings support that the dominant learning style is not exclusively an essential factor to predict educational achievement. Rather, it shows learning preferences of students that may be considered in designing learning opportunities by the teachers.

## 1. Introduction

In recent decades, the concept of individuals’ differences in learning has been the subject of many studies (Saif, 2005). The validity of an educational institute is dependent on learners’ performance level. Identifying factors that influence learning can resolve problems and defects in an educational program. One of these factors is learning style (Shah, Ahmed, Shenoy, & N, 2013). Based on individual differences, students use different learning styles. Saif states that some educational psychologists, including Woolfolk, prefer the term “Learning Preference” rather than “Learning Style” (Saif, 2005). Curry believes that differences between students’ educational achievement is more related to their adaptation to learning style with the environment and assignments rather than their general abilities (Curry, 1999).

**Figure 1:**
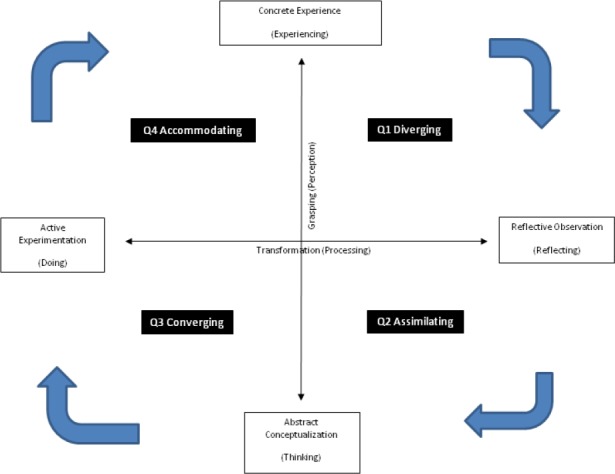
Learning Cycle based on Kolb Experiential Learning Theory

Hitherto, various classifications of learning styles have been presented. One of these classifications has been introduced by David A. Kolb based on his theory about experiential learning. He believes that learning takes place in a four-stage cycle (Figure 1). Firstly, learner encounters a topic, content or learning situation (Concrete Experience); then, observes and reflects upon it (Reflective Observation); afterwards, the learner starts to think, grasp the concept and summarize his/her opinions and perceptions (Abstract Conceptualization); and finally, begins to experiment (Active Experimentation) (Kolb, Boyatzis, & Mainemelis, 2000). However, all learners do not have similar abilities in all stages of the cycle. Each person has his/her own special interests and preferences that lead to different learning styles, and consequently, they select different majors of study or careers. In this regard, Kolb designed a self-assessment tool in which, individual’s learning preferences or styles are determined according to the scores and the characteristics of the graph drawn based on the scores. He mentions four learning styles including Assimilating, Accommodating, Converging, and Diverging. For instance, learners who tend to reflective observation and abstract conceptualization have Assimilating learning style (Kolb et al., 2000).

Yet, several studies on Kolb learning styles have been conducted among students of different majors including medicine in Iran (Ghaffari et al., 2013; Meyari, Sabouri Kashani, Gharib, & Beiglarkhani, 2010; Salehi, Soleimani, Amini, & Shahnoushi, 2000; Sarchami & Hossaini, 2004; Valizadeh, Fathi azar, & Zamanzadeh, 2006). Saif and Hosseini Lorgani carried out a study on students from different majors and acknowledged that most of medical students’ learning style is Assimilating (Saif & Hosseini Lorgani, 2001). Moreover, in Azizi, Khanzadeh and Hosseini study on medical students, the dominant learning styles were Assimilating and Converging respectively (Azizi, Khanzadeh, & Hosseini, 2002). Although several studies have been done on Kolb learning styles and its relation with variables such as educational achievement, there are some critics that question the theory and its practical usage to identify individuals’ learning styles. Kayes argues over the importance of language and discourse in learning process which is not considered in this theory (Kayes, 2002). Also, Hopkins believes that Kolb does not explain the nature of experiential process (Hopkins, 1993); his theory is popular due to being easy to use (Armstrong & Parsa-Parsi, 2005). However, based on this theory, several studies have been done on different learners all around the world (Alcota, Muñoz, & González, 2011; ALQahtani & Al-Gahtani, 2014; Bitran, Zúñiga, Pedrals, Padilla, & Mena, 2012; Fang, 2002; Ghaffari et al., 2013; Meyari et al., 2010).

The field of dentistry is combined of theory and practice; therefore, it is highly important to recognize students’ learning preferences in order to design an effective educational curriculum and provide learning opportunities. Fang has conducted a survey of pre-doctoral students at Columbia University, School of Dental and Oral Surgery, applying different theories including Kolb’s theory, to obtain data on different learning styles of dental students and their preferred teaching methods. The results presented that curriculum changes can be developed to provide an optimal learning experience for students (Fang, 2002). Alcota et al. carried out a study to identify the learning styles of a group of low marks dental students in Chile to improve their educational achievement by means of remedial teaching. After educational intervention, the mean of the scores obtained by the group were higher than the average scores before intervention. They concluded that identifying the students’ learning styles can help the teachers to design educational methods more effective (Alcota et al., 2011).

ALQahtani and Al-Gahtani conducted a study to identify learning styles among Saudi dental students and interns utilizing Kolb’s Learning Style Inventory (LSI). The results showed that the Diverging learning style was the dominant style. Moreover, no associations between students’ learning style and their gender, GPA, or specialty interest was seen (ALQahtani & Al-Gahtani, 2014).

In Iran, although several studies utilizing Kolb’s theory have been conducted among different fields of medical sciences (Azizi et al., 2002; Ghaffari et al., 2013; Meyari et al., 2010; Saif & Hosseini Lorgani, 2001; Salehi et al., 2000; Sarchami & Hossaini, 2004; Valizadeh et al., 2006); few studies have been done to identify learning preferences on dental students. The purpose of this research was to determine the educational achievement among dental students in relation to their dominant learning styles based on Kolb theory in Mashhad University of Medical Sciences through 2010-2011.

## 2. Method

The study participants were selected via purposive sampling from dental students of Mashhad University of Medical Sciences that had passed Comprehensive Basic Sciences Exam (CBSE) and were studying in second semester of 2010-2011. A list of the students was obtained from the educational services office. One of the researchers coordinated the teachers to explain the survey in the classes to assure the students about the confidentiality and take their written informed consents. The research tools, including demographic questions and Kolb Learning Style Inventory (Ver. 3.1) were administered on the sample. The validity and reliability of the original questionnaire was approved by Kolb and his colleagues (Kolb et al., 2000). Also, the validity and reliability of Persian version of the questionnaire had been shown in several studies in Iran (Saif & Hosseini Lorgani, 2001; Sarchami & Hossaini, 2004; Valizadeh et al., 2006). The questionnaire had 12 statements, each with four choices. The participants should rank the choices from 1 to 4 according to their preference. The scores of individual orientations to each learning styles were ascertained according to respective benchmarks and charts. A total of 215 students were qualified to participate in our study. However, 182 of them accepted to complete the questionnaire and signed a written consent, and 20 questionnaires were extracted due to defect, distortion, lack of attention to rank the choices. Research variables related to students’ educational achievement such as GPA, theoretical courses marks, practical courses marks, and CBSE mark, were extracted from database and archive of educational services office. We used χ^2^, Kolmogorov-Smirnov, and two-way ANOVA tests to analyze the data, using SPSS 16.

## 3. Results

**Figure 2:**
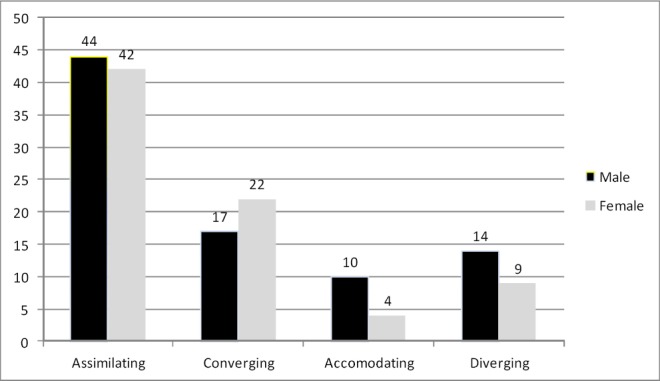
Distribution of participants’ learning style according to gender and number

Most of the students were male (52.5%). The mean for age was 24.3±5.5 years. Seventy six percent of the participants were single and 24.1% were married. Most of the participants’ dominant learning styles were Assimilating (53.1%). The frequency distributions of learning styles by gender are presented in Fig. 2.

There was no significant relationship between learning style and gender according to χ^2^ test (P = 0.266, df = 3). Moreover, there was no significant relationship between learning style, age and marital status of the participants (P≥0.05). Also, the relationship of learning styles and students’ educational achievement variables such as GPA, theoretical courses marks, practical courses marks and CBSE mark were assessed. Firstly, Kolmogorov-Smirnov test was carried out to ensure normal distribution of quantitative variables. As the variables were not distributed normally, Kruskal-Wallis test was performed to determine the relationship between learning styles and students’ educational achievement variables. Table 1 compares the scores obtained by students with different learning styles.

**Table 1:** The relationship between learning style and students’ academic achievement variables

Learning Style	Diverging	Accommodating	Assimilating	Converging	P Value
GPA	15.14±0.28	15.17±0.39	15.89±0.16	15.67±0.22	0.079
Average of theoretical courses marks	14.62±0.30	14.62±0.42	15.46±0.19	15.18±0.25	0.079
Average of practical courses marks	16.23±0.28	16.34±0.33	16.89±0.12	16.84±0.14	0.083
CBSE mark	126.82±4.63	128.86±7.51	129.68±3.09	125.53±4.37	0.781

The results showed that there was a statistically significant relationship between students’ gender and marks of educational achievement. In other words, female students’ average marks of educational achievement were higher than males. Also, there was no significant relationship between learning styles and variables of educational achievements. Therefore, we decided to assess the interaction between learning styles and gender on educational achievements variables. The results of two-way ANOVA test showed that there was no statistically significant relationship between these factors.

## 4. Discussion

In this study male and female students were not different in their learning styles, which was similar to the results of other studies (AL-Qahtani & Al-Gahtani, 2014; Ghaffari et al., 2013; Meyari et al., 2010; Saif & Hosseini Lorgani, 2001). Moreover, this study showed that most of the participants’ learning style was Assimilating (53.1%); which is concordant with the findings of studies conducted on medical students (Azizi et al., 2002; Ghaffari et al., 2013; Saif & Hosseini Lorgani, 2001). Similarly, in Alcota et al.’ study regarding good and weak dental students’ learning styles, the results represented that dominant learning style of good students was Assimilating (34%) (Alcota et al., 2011). In a study performed by ALQahtani and Al-Gahtani, dental students’ dominant learning style was Diverging, though, the students learning style was Assimilating before clinical course, and tend to become Diverging in the following years (ALQahtani & Al-Gahtani, 2014). This may be due to cultural differences. ALQahtani and Al-Gahtani used the original English version of Kolb learning style questionnaire while we used the Persian translated one in present study. Some experts have criticized that Kolb theory takes very little account of different cultural experiences and conditions. Smith quotes from Anderson that Kolb questionnaire has been used in few different populations, which mainly were western societies and there is a need to consider the culturally-based differences in cognitive and communication styles. He believes that the different models of selfhood, and the extent of difference from western culture should be considered in this kind of studies (Smith, 2010).

Kayes refers to theories such as Vygotsky’s social learning theory that emphasizes on the fundamental role of social interaction in the process of cognitive development. He, quoting from Holman, criticizes the individual nature of learning in Kolb theory (Kayes, 2002). However, Kolb believes that Assimilating learners appear more willing to conceptualize and reflect on theories; they are capable to retain and process an enormous amount of information. Therefore, it seems Assimilating learning style is more appropriate for dental curriculum especially in pre-clinical period. This study showed that many students had a tendency to Converging learning style (24.1%). Converging learners are more capable to provide feasible solutions and implement ideas. Actually, these people are interested to work in laboratories and practical applications (Kolb et al., 2000). It seems that the nature of dentistry is more congruent with this learning style, especially in practical courses and during internships.

An important point in our results was the relationship between learning style and educational achievements variables. Although, Kruskal-Wallis test did not show any significant relationship between learning style and GPA (P=0.079), theoretical courses marks (P=0.079), practical courses marks (P=0.083) and CBSE mark (P=0.781); these educational achievement variables were higher in Assimilating and Converging students than Accommodating and Diverging groups (Table 1). This is congruent with Kolb experiential learning theory, considering the nature of courses and assignments in dentistry education. Assimilating learners are more capable in theoretical courses, while Converging students are better in practical courses. However, differences in performance were not much noticeable in our study. In other words, even Accommodating and Diverging learners’ performances were similar to others. Our results are in line with ALQahtani and Al-Gahtani’s study carried out on dental students (ALQahtani & Al-Gahtani, 2014), and Sarchami and Hossaini’s study conducted on nursing students (Sarchami & Hossaini, 2004) which showed no relationship between four learning styles and GPA. In Alcota et al.’s study, 54% of good dental students were Assimilating (34%) and Converging (20%) learners, while the weak students mostly had dominant Diverging (54.2%) and Accommodating (14.3 %) learning styles (Alcota et al., 2011).

In a study conducted by Al Saud regarding the association between learning style preferences of a group of first-year dental students and GPA, the VARK questionnaire was used to determine the students’ preferred mode of learning. He realized that students with a single learning style preference had a lower mean GPA than those with multiple (quad-modal) learning style preferences (Al-Saud, 2013). Also, another study using VARK questionnaire, reported that dental students preferred multimodal learning style (Shah et al., 2013). Similarly, according to Kolb learning theory, the perfect learner may be the one who can appropriately use different learning styles in different situation and topics (Kolb et al., 2000). Many researchers believe that if teachers know the application of learning styles, they will recognize the learners better in order to provide learning opportunities using various methods, and to develop the efficiency of educational curriculum for everyone. Alcota and his colleagues reported considerable findings after utilizing diverse and collaborative teaching methods to develop weak students’ performances (Alcota et al., 2011). Fang also suggests that identifying dental students’ learning styles is necessary to initiate changes in traditional curriculum and make them more flexible. There should be less lecture-based programs, while mentorship relationship between students and teacher must be provided to prepare them for clinical fields (Fang, 2002). Similarly, Al-Saud states that dental educators need to apply variety of presentation styles to create more effective learning environments for all students (Al-Saud, 2013).

Armstrong and Parsa-Parsi state that promoting the application of all learning abilities based on Kolb cycle sequentially by the learners, is the most desirable approach in an educational encounter. However, it does not mean that teachers should adopt their teaching method with each learner’s learning style; rather using Kolb learning cycle, they should provide situations for students to apply their four modes of preferences effectively. They employed this framework in the professional development courses designed for physicians seeking to develop their skills as leaders of educational programs in multiple health care environments at Harvard University. For instance in Q1 (Fig.1), it is important to activate prior and current knowledge of learners in relation with the course via bringing questions, goals, issues, and real-world projects that set the stage for learning to be anchored in tasks and goals the learner has defined. In Q2, the lectures and journal clubs can be used to bring new information, data, and principles for the learner to enrich and expand the existing fund of knowledge required to address the learner’s needs or meet his or her goals. In Q3, Kolb’s third quadrant calls for a shift from thinking to acting, moving from theory to practice. Preparation for a case-discussion requires individuals to analyze a problem scenario and come to class ready to summarize their assessment and proposed solution. Moreover, it is worthwhile to use simulation in clinical skills centers and laboratories in this stage. The turning point is that the learner becomes fully committed to use the skill or content in the learned subjects. In Q4, the learner is committed to put a new behavior into practice in his/her own setting. This new behavior can become the baseline for repeating the learning cycle and following sequential stages (Armstrong & Parsa-Parsi, 2005).

According to the results, it seems that learning style is not an essential factor for learners’ achievement in an educational curriculum; rather, there are other influential factors, such as motivation. Saif and Hosseini Lorgani, quoting from Ormrod, argued that cognitive and learning styles are greatly cultural-based, and are less stable than intellectual abilities, and can be modified during time. To set an example, the question is whether teamwork is a social value orientation of the person or not (Saif & Hosseini Lorgani, 2001). In a longitudinal survey of a group of medical students in Pontificia Universidad Católica de Chile (PUC) by Bitran and his colleagues, the results revealed that students’ learning styles were modified during time. In the first year, the dominant learning style of students was rather Assimilating (54 %) and Converging (26 %), which did not change till the third year. However, in the seventh year, 49% of the students had Converging style followed by Assimilating (33%). They suggest this change might represent an adaptation to the curriculum and teaching methods. Generally, in the first years of the dental educational program, the curriculum is mostly based on lecture and teacher-centered model. Although, in the next years, it is changed to a problem-based student–centered model (Bitran et al., 2012). As Hopkins suggests, this might represent the instability of Kolb learning style in different situations. Therefore, students should not be labeled (Hopkins, 1993). Saif, quoting from Kolb, alludes to the flexibility of learning styles and states each learning style has its own strengths and weaknesses; therefore, a perfect learner is not the one that uses only one style. The student should be able to use different learning styles in different situations (Saif, 2005). Kolb and his team attempted to revise Kolb Learning Style Inventory (Ver 3.1) to clarify ambiguities and criticism on the four-class category. They developed a detailed nine-class category for learning styles. One of them is “Balancing” and the others are according to learners’ preference and can be determined using Kolb Learning Style Inventory (Ver. 4) (“Kolb Learning Style Inventory (LSI) Version 4,” 2014). This new categorization can be the basis of future researches.

Finally, although experiential learning theory has met criticism, it helps, to some extent, to recognize individual differences in learners. Moreover, teachers of different majors, including dentistry, can enhance their students’ motivations through relating new knowledge with prior knowledge, presenting course materials differently, encouraging them to think and experiment the materials, in order to deepen students’ learning
